# Simultaneous Quantification of Alcoholic Content and Acidity in Brazilian Commercial Kombuchas by FTIR Spectroscopy and Chemometric Analysis

**DOI:** 10.1111/1750-3841.70433

**Published:** 2025-08-03

**Authors:** Gabriela Fioravante da Silva, Bruno Gonçalves Botelho

**Affiliations:** ^1^ Chemistry Department ICEx, Federal University of Minas Gerais Belo Horizonte Minas Gerais Brazil

**Keywords:** acidity, alcoholic content, chemometric models, kombucha quality control, validatio

## Abstract

**ABSTRACT:**

The growth of the global kombucha market highlights the importance of quality control in production, a crucial factor to ensure consumer safety, regulatory compliance, and product consistency. This study aimed to develop and validate rapid, direct multivariate methods based on Fourier transform infrared (FTIR) spectroscopy combined with partial least squares (PLS) regression to simultaneously quantify alcohol content and acidity. The method requires minimal sample preparation, with no need for extraction or derivatization, allowing spectral acquisition in about 30 s—ideal for high‐throughput analysis and aligned with green chemistry principles. Infrared spectra were recorded in the range of 4000–650 cm⁻¹. Reference values were obtained by gas chromatography with flame ionization detection (GC‐FID), following validated protocols. Thirty kombucha brands from different regions of Brazil were analyzed, all labeled with an alcohol content of up to 0.5% v/v. However, 60% of the samples exceeded this limit, with levels ranging from 0.10% to 1.70% v/v. Acidity ranged from 17 to 71 mEq/L, with only 50% of the samples meeting the recommended specifications (30–130 mEq/L). The PLS model showed RMSEC and RMSEP values of 0.09% and 0.07% v/v for alcohol, and 3.2 and 3.9 mEq/L for acidity, respectively. Metrics such as precision, bias, and RPD > 2.4 confirmed the robustness of the models. The results reveal discrepancies between declared and measured values, especially for alcohol content, emphasizing the need for stricter regulatory oversight. The method is reliable, efficient, and suitable for routine analyses and regulatory monitoring.

**Practical Applications:**

The FTIR‐PLS method developed in this study offers a rapid, accurate, and environmentally friendly alternative for routine monitoring of alcohol content and acidity in kombucha. Its application facilitates verification of regulatory compliance and strengthens quality control in the beverage industry, contributing to consumer safety. Moreover, the method can be adopted by regulatory agencies and producers to ensure the conformity of products available on the market.

## 1 Introduction

A growing interest in functional foods and wellness products has highlighted kombucha as an alternative to soft drinks and fermented dairy beverages. Kombucha is a fermented beverage produced from sweetened tea fermented by a symbiotic culture of bacteria and yeast (SCOBY) (Brasil 2019; Villarreal‐Soto et al. 2018). Originating in China over 2000 years ago, its popularity expanded in Europe during the 1960s, and it is now consumed worldwide (Miranda et al. 2022). This beverage is recognized for its antioxidant, antimicrobial, hypoglycemic, detoxifying, and probiotic properties, although in Brazil it is not yet officially classified as a probiotic, requiring further studies for confirmation (Watawana et al. 2015; Tran et al. 2022).

The global kombucha market has shown remarkable growth in recent years. In 2024, the sector was estimated at around $4.26 billion, with projections to reach approximately $9.09 billion by 2030, representing a compound annual growth rate (CAGR) of 13.5% between 2025 and 2030 (Grand View Research 2024). In the United States, the leading market, revenue in 2023 was about $1.33 billion, with expectations to reach $4.94 billion by 2032, driven by a CAGR of 15.7% from 2024 to 2032. In Brazil, although the market is still in its early stages, significant progress has been made. The establishment of the Brazilian Kombucha Association (ABKOM) in 2018 marked an important step in organizing the sector, currently bringing together around 30 producers and promoting the regulation and standardization of product quality. According to the *Non‐alcoholic beverages yearbook* (MAPA 2024), the number of kombucha factories in the country grew by 923.5% between 2019 and 2023, driven by digital marketing strategies, especially on social media, as well as innovation in flavors and packaging, factors that have increased consumer interest.

Quality control in kombucha production is essential to ensure consumer safety, regulatory compliance, and consistency of the final product. Two key parameters for evaluation are alcohol content and acidity. Acidity, mainly attributed to acetic acid, plays an important role in inhibiting microbial growth and contributing to the characteristic flavor of the beverage. Alcohol content is a crucial indicator of fermentative activity and must be rigorously monitored to keep the process within established limits, avoiding excessive alcohol formation (Villarreal‐Soto et al. 2019; Al‐Mohammadi et al. 2021). The balance between acidity and alcohol content directly influences consumer acceptance, and maintaining consistency in these parameters across batches is essential to build trust and brand loyalty.

In Brazil, Normative Instruction No. 41, dated September 17, 2019, requires alcohol content to be indicated on kombucha labels. For non‐alcoholic versions, the permitted limit is up to 0.5% v/v, with a tolerance of 0.1% v/v, and beverages labeled as “zero alcohol” can contain up to 0.05% v/v. For alcoholic kombucha, alcohol content may range from 0.6% to 8.0% v/v (Brasil 2019). This variety reflects product diversity, offering consumers different choices according to their preferences. Transparency in alcohol content is crucial for informed choices, especially for vulnerable groups such as children, pregnant women, and individuals abstaining for health, religious, or legal reasons. Regarding acidity, it must be declared based on volatile acidity, which corresponds to low molecular weight acids that volatilize upon heating, mainly acetic acid, with recommended values between 30 and 130 mEq/L (equivalent to 0.17%–0.74% v/v) (Brasil 2019).

Various techniques are employed to determine alcohol content in beverages, including kombucha, such as gas chromatography (Ebersole et al. 2017; Talebi et al. 2017; Chan et al. 2021), enzymatic assays (Lacorn and Hektor 2018), and distillation followed by densitometry (Ortiz et al. 2022), the latter standing out for simplicity and not requiring complex equipment. For acidity assessment, acid‐base titration is the most common technique due to its simplicity and low cost, as demonstrated by several studies (Suhre 2020; Dada et al. 2021; Hume 2021; Cabral 2021). Most of these focus on total acidity quantification; however, volatile acidity determination requires a preliminary distillation step before titration. Other techniques, such as high‐performance liquid chromatography, are also used (Villarreal‐Soto et al. 2019; Santos 2020).

With the global increase in commercialization and consumption of kombucha, there is rising demand for rapid and simple analytical techniques for quality control. In recent years, mid‐infrared (MIR) and near‐infrared (NIR) spectroscopy combined with chemometrics have gained popularity in food and beverage analysis, offering advantages over traditional methods like chromatography. These techniques provide rapid results, with spectra collected in seconds depending on equipment configuration (spectral resolution and scan number), allowing analysis of many samples in short time. Moreover, sample preparation is minimized, often eliminating extraction, dilution, or derivatization steps, reducing time and cost. Another benefit is the ability to analyze liquid or solid samples directly with minimal lab intervention, facilitating automation and real‐time monitoring in the food industry (Santana et al. 2020; Souza et al. 2013). From a green chemistry perspective, MIR/NIR combined with chemometrics is a significant advance, as it eliminates or reduces toxic reagents, minimizing waste, exposure to hazardous substances, and environmental impact. Besides improving industrial process efficiency, these approaches align with sustainable practices, contributing to safer, more economical, and environmentally responsible production (Ballesteros‐Vivas et al. 2021).

The application of chemometric methods in kombucha analysis is still underexplored, with most studies using samples prepared for research purposes (Zhao et al. 2023; Wang et al. 2023; Barbosa et al. 2020). This work used commercial samples from different Brazilian manufacturers and regions, providing greater robustness and representativeness to the developed models. The objective of this study was to develop chemometric models based on MIR spectroscopy and partial least squares (PLS) regression to simultaneously quantify alcohol content and volatile acidity in kombucha. The approach aims to offer a fast and simple solution for analyzing these parameters, reducing sample preparation and allowing evaluation of a large volume of samples. Thus, the study seeks to provide clear and transparent information to consumers regarding product composition in the Brazilian market, assessing compliance with current regulations, as well as facilitating quality control and ensuring regulatory conformity. Reference values were obtained using gas chromatography with flame ionization detection (GC‐FID). Both the multivariate and reference methods were validated according to national and international guidelines to ensure reliability and suitability.

## 2 Materials and Methods

### 2.1 Instruments and Software

The infrared spectra of the kombucha samples were acquired using a Fourier transform infrared (FTIR) spectrophotometer (Frontier model, PerkinElmer, Waltham, MA, USA). An attenuated total reflectance (ATR) accessory with a diamond crystal was employed. The spectra were recorded in transmittance mode within the range of 4000–650 cm⁻¹, with a resolution of 4.0 cm⁻¹ and a total of 32 scans. The transmittance versus wavenumber measurements were collected and organized into an Nxp data matrix. In this matrix, the rows represent the *n*‐samples (96), and the columns represent the *p*‐variables (wavenumbers, 3100). Additionally, an ynx1 vector was constructed containing the analyte concentrations. Multivariate calibration models using PLS were developed using the MATLAB software (version R2010a, MathWorks, USA), in conjunction with the PLS Toolbox (Eigenvector Research, USA).

### 2.2 Samples

A total of 96 commercial kombucha samples from 30 brands located in the states of São Paulo, Goiás, Distrito Federal, Minas Gerais, Bahia, Roraima, Paraná, Rio de Janeiro, Pernambuco, and Rio Grande do Sul were analyzed. All samples declared an alcohol content of up to 0.5% v/v on the label. The collections took place between 2021 and 2022, with the products sent to the laboratory by the manufacturers themselves for quality assessment. The samples included both the original version and flavored variations. The study was registered in the National System for the Management of Genetic Heritage and Associated Traditional Knowledge (SISGEN‐Brazil) under the number AFA583E.

### 2.3 Reagents

The working solutions were prepared with high‐purity reagents, namely, ethanol (CAS 64‐17‐5, Sigma‐Aldrich), acetic acid (CAS 64‐19‐7, Sigma‐Aldrich), and 1‐butanol (CAS 71‐36‐3, Sigma‐Aldrich). Deionized water (18.2 MΩ cm⁻¹ at 25°C) was obtained from a MilliPore Direct‐Q 3 UV system, manufactured by Merck Millipore (France).

### 2.4 Sample Preparation

The kombucha samples were degassed, and 200 mL were transferred to 250 mL Erlenmeyer flasks, with the addition of two drops of simethicone (75 mg/mL), and stirred for 15 min in an orbital shaker (Kacil brand, model HM01) until the bubbles ceased. The samples were then stored at −18°C until the time of analysis. Before testing, the samples were thawed at room temperature (25°C), centrifuged at 5000 rpm for 5 min in a centrifuge (KASVI, model K 14–0815P) to remove suspended particles, and divided for reference and chemometric methods. For the GC‐FID method, 250 µL of the supernatant were mixed with 250 µL of water and 20 µL of 5% v/v 1‐butanol, and the tubes were vortexed for 10 s at 3000 rpm. For the chemometric models, 50 µL of the supernatant were pipetted directly into the ATR cell, which was cleaned with cotton and acetone before each reading.

### 2.5 Reference Method GC‐FID

A method was developed and validated using GC‐FID for the simultaneous quantification of alcohol content and volatile acidity in kombucha samples. The choice of GC‐FID was due to its high sensitivity, selectivity, excellent reproducibility, and wide linear range, characteristics that make it particularly effective for analyzing volatile compounds such as ethanol and short‐chain organic acids, even in complex matrices (Skoog et al. 2009). The proposed method enabled the simultaneous determination of both analytes in a single chromatographic run, with simple sample preparation and direct injection, optimizing both time and analytical resources. Analyses were performed on a Shimadzu gas chromatograph, model GC‐17A (Kyoto, Japan), equipped with a flame ionization detector (FID) and a SUPELCOWAX 10 polar capillary column (30 m × 0.53 mm × 2.0 µm; Supelco, Bellefonte, PA, USA) coated with polyethylene glycol (PEG). High‐purity hydrogen (99.999%) was used as the carrier gas at a flow rate of 2.6 mL/min. The injector and detector temperatures were maintained at 240°C. A 0.5 µL aliquot of each sample was injected in split mode (1:10), and separation was achieved in 11.3 min with the following temperature program: starting at 120°C, ramping at 20°C/min to 170°C (held for 0 min), then 5°C/min to 195°C (0 min), and finally 20°C/min to 230°C, with a final hold of 2 min. The reference method was validated according to national (INMETRO 2020) and international (EURACHEM 2025) guidelines, evaluating linearity, matrix effects, detection and quantification limits, recovery, and precision.

### 2.6 Analytical Validation

#### 2.6.1 Linearity and Matrix Effect

To evaluate linearity, three independent analytical curves were prepared with concentrations of 0.1%, 0.2%, 0.3%, 0.4%, 0.5%, and 0.6% v/v of ethanol and acetic acid in deionized water. The working range of 0.1%–0.6% v/v corresponds to 17–105 mEq/L of acetic acid. The matrix effect was evaluated by comparing calibration curves constructed in solvent (deionized water) and in a kombucha matrix with low analyte content, using the same concentration levels. Statistically significant differences in the slopes of the two curves (*p* < 0.05, ANOVA) indicated the presence of a matrix effect for both ethanol and acetic acid. In accordance with EURACHEM (EURACHEM 2025) and INMETRO (INMETRO 2020) guidelines, this result required all further validation parameters—including detection limits, quantification limits, precision, and recovery—to be determined using matrix‐matched samples rather than solvent‐based standards.

#### 2.6.2 Detection Limit and Quantification Limit

The limit of detection (LOD) and quantification (LOQ) were determined using 10 independent blanks, with the process kombucha as the matrix, prepared in the same manner as the samples, as described in Section 2.4.

#### 2.6.3 Recovery and Precision

Recovery was assessed by adding known amounts of the analytes to the matrix at three levels (0.1%, 0.3%, and 0.6% v/v), with six replicates per level. Precision was determined through repeatability (on the same day and operator) and intermediate precision (on different days), with results expressed as relative standard deviations and analyzed by ANOVA, after confirming normality, homoscedasticity, and independence of the residuals.

#### 2.6.4 Multivariate Analytical Validation

The analytical validation of multivariate calibration methods is still not a fully established topic. This study combined univariate and multivariate concepts, adapting Brazilian and international guidelines to estimate parameters such as linearity, working range, precision, bias, and the ratio of performance to deviation (RPD) (INMETRO 2020; EURACHEM 2025; Fulgêncio et al. 2022; Botelho et al. 2013).

### 2.7 Statistical Analysis

The data were assessed for normality using the Shapiro–Wilk test, yielding a *p*‐value > 0.05, indicating compliance with the normality assumption. Descriptive measures such as mean, median, and standard deviation, among others, were calculated and are presented in Table S1. Statistical analyses were performed using OriginPro version 8.5 (OriginLab Corporation, USA).

## 3 Results and Discussions

### 3.1 Reference Method GC‐FID

The chromatographic method demonstrated robustness in the analysis of Kombucha samples, standing out for its simplicity, speed, and minimal sample preparation. The incorporation of an internal standard (1‐butanol) significantly contributed to compensating for instrumental variations and improving the precision of the results.

**FIGURE 1:**
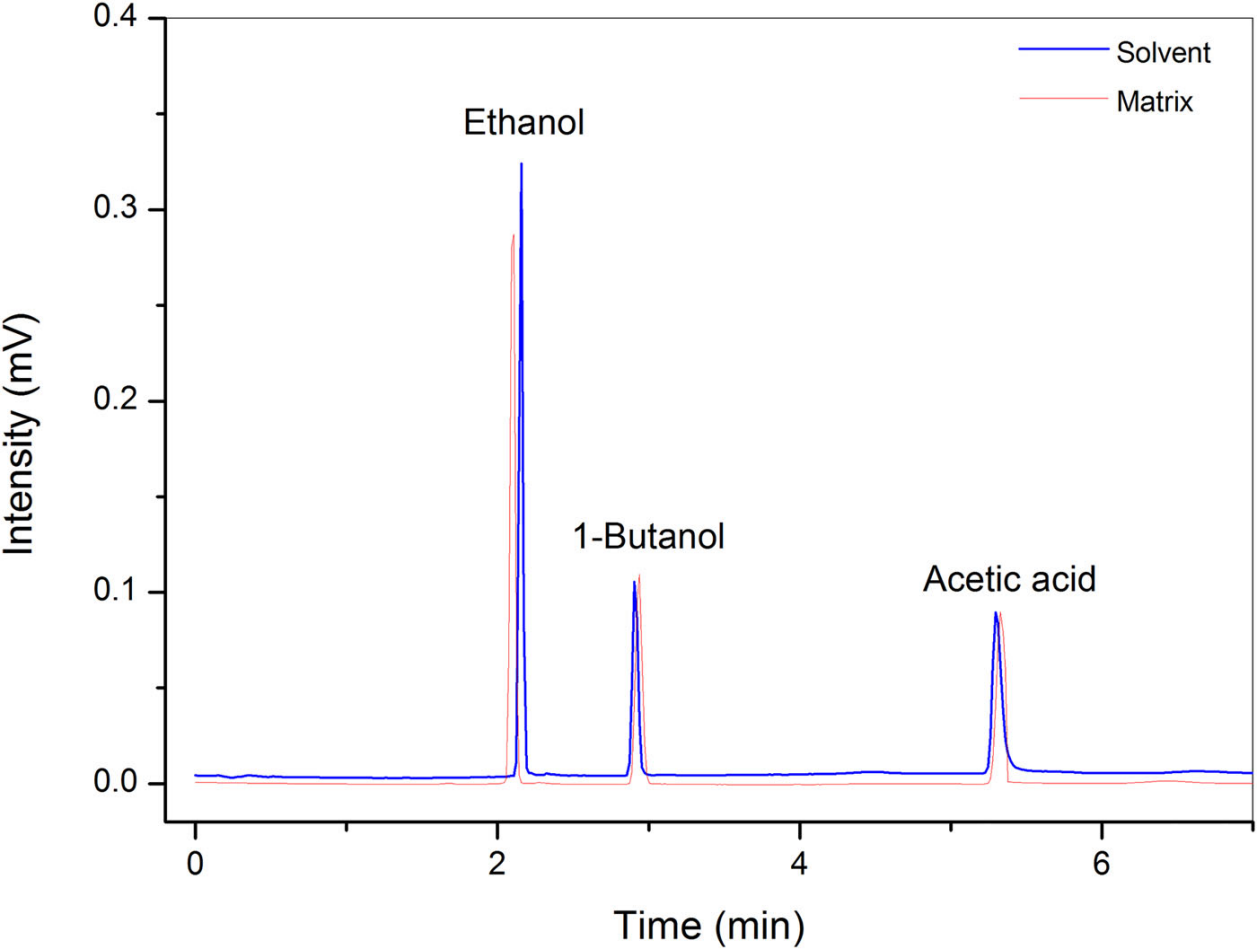
Chromatogram of the mixed standard (0.6% volume per volume [v/v] ethanol and acetic acid) in matrix (red) and in solvent (blue).

The chromatogram in Figure 1 illustrates effective resolution between ethanol, acetic acid, and the internal standard (1‐butanol), with no peak overlap, confirming the method's selectivity. The retention times were 2.11 min for ethanol, 5.16 min for acetic acid, and 2.92 min for 1‐butanol, with relative standard deviations (RSD) below 1.5% across six replicates, indicating good repeatability. The resolution (Rs) values between adjacent peaks were above 2.0, exceeding the minimum criterion (Rs > 1.5) for baseline separation. The use of 1‐butanol as an internal standard allowed for correction of injection volume variability and detector fluctuations, improving quantitative consistency.

The calibration curves constructed with and without internal standard correction showed an increase in *R* ^2^ from 0.9741 to 0.9930 for ethanol and from 0.9843 to 0.9916 for acetic acid, confirming the positive effect of the internal standard on the method's precision. The choice of the SUPELCOWAX 10 chromatographic column proved to be appropriate due to its PEG stationary phase, which favors selective interactions with polar compounds, ensuring effective separation of the analytes.

The choice of the SUPELCOWAX 10 chromatographic column proved to be appropriate due to its polar PEG stationary phase, which favors selective interactions with polar compounds, ensuring effective separation of the analytes.

The optimized methodology enables simultaneous monitoring of alcohol content and volatile acidity (as acetic acid), which are essential parameters in the quality control of Kombucha.

### 3.2 Analytical Validation of the Reference Method

The linearity of the calibration curves was evaluated both in solvent and in matrix. Outliers were removed using the Jackknife standardized residuals test, with a maximum exclusion criterion of 22.2%. Three outliers were excluded from the ethanol curve in solvent and three from the matrix, along with two from the acetic acid curve in solvent, with no exclusions in the matrix curve. The calibration curves, after the removal of outliers, are presented in Figures S1 and S2.

The linear regressions of the calibration curves were obtained using the ordinary least squares (OLS) method. The normality, independence, and homoscedasticity of the residuals were verified using the Ryan–Joiner, Durbin–Watson, and Brown–Forsythe tests, respectively. The analyses confirmed that the residuals were normally distributed, independent, and homoscedastic (*p* > 0.05 for all tests), with significant differences observed in the slopes between the solvent and matrix curves (*p* < 0.05 in the slope comparison, *t*‐test), indicating a matrix effect for both analytes.

The limit of quantification (LOQ) was defined as 0.1% v/v for both analytes, as this was the lowest level at which the method's trend, recovery, and precision were evaluated. The LOD was calculated as 0.03% v/v, using the equation LOD = LOQ/3.3.

The repeatability values ranged from 2.0% to 3.4%, intermediate precision ranged from 2.3% to 3.6%, and recovery ranged from 96% to 104%, all in accordance with the acceptance criteria of the AOAC (Association of Official Analytical Chemists), an international organization that develops and publishes standard analytical methods and quality criteria to ensure the precision and reliability of analytical results (AOAC 2016). A summary of the merit figures is presented in Table 1.

**TABLE 1:** Summary of merit figures for the validation of the method for ethanol and acetic acid.

Parameter	Ethanol	Ácetic acid
Working range LOD	0.1%–0.6% v/v 0.03% v/v	0.1%–0.6% v/v (17–105 mEq/L) 0.03% v/v (6 mEq/L)
LOQ RSD repeatability (%) RSD intermediate precision (%) Recovery (%)	0.1% v/v 2.0–3.4 2.7–3.6 96–101	0.1% v/v (17 mEq/L) 2.3–3.2 2.3–3.5 100–104

Abbreviations: LOD = limit of detection; LOQ = limit of quantification; Recovery (%) = percent recovery; RSD intermediate precision (%) = relative standard deviation for intermediate precision; RSD repeatability (%) = relative standard deviation for repeatability; Working range = working range.

After validation, the method proved to be efficient in the simultaneous determination of both parameters, requiring little sample preparation, demonstrating good precision, and having low detection limits.

### 3.3 Application of the Validated Method for Determining the Alcohol Content and Acidity of Kombucha Samples by GC‐FID

**FIGURE 2:**
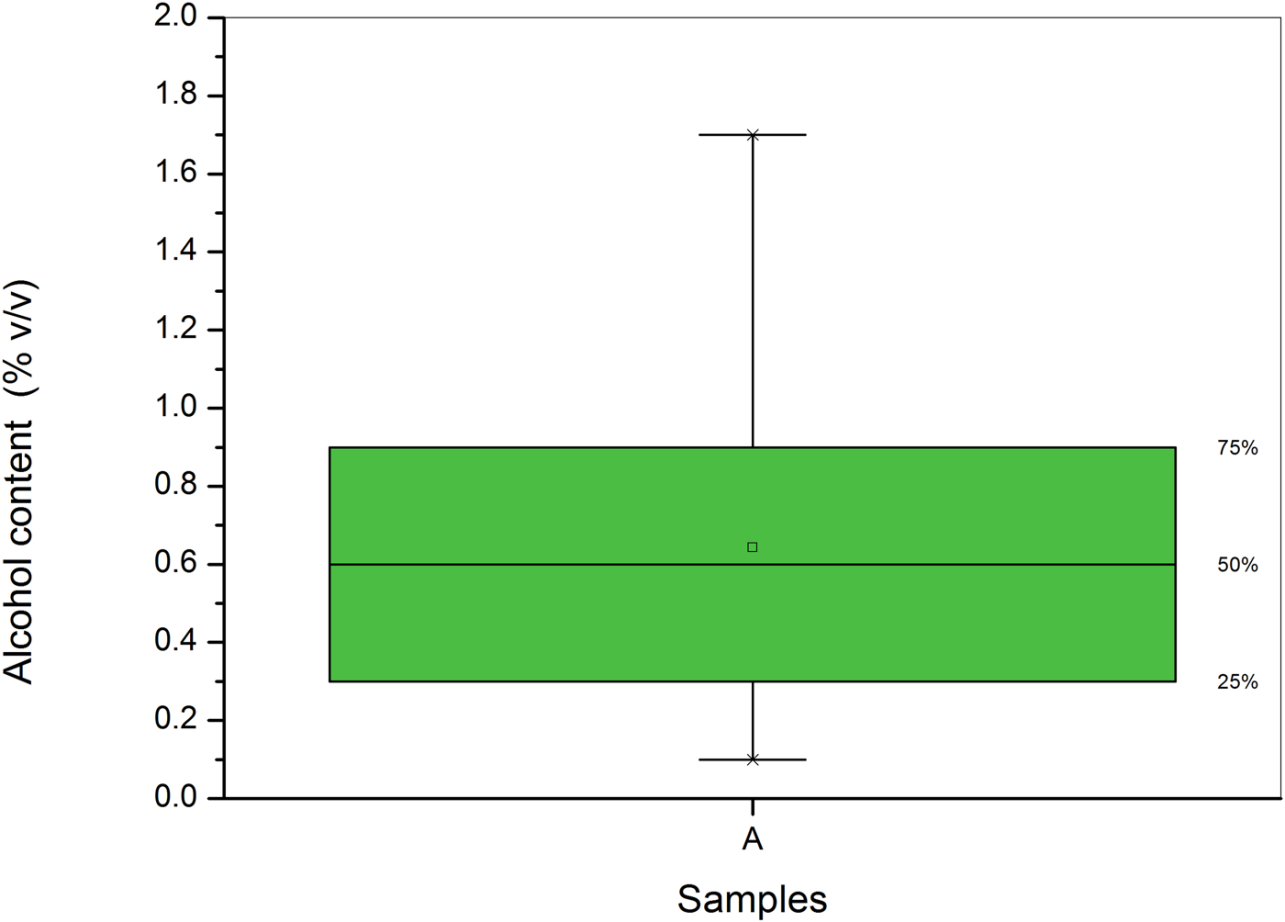
BoxPlot of the alcoholic content (percentage volume per volume [% v/v]) of commercial kombucha samples with values above the method's limit of quantification (LOQ). The square represents the median.

The analyzed kombucha samples showed ethanol content ranging from 0.10% to 1.70% v/v, with some requiring dilution for analysis. Fifty percent of the samples had concentrations between 0.3% and 0.9% v/v, as shown in the boxplot graph in Figure 2. Only 38 of the 96 samples labeled as non‐alcoholic complied with the legislation (< 0.5% v/v), highlighting the need for rigorous analytical controls. No outliers were identified.

**FIGURE 3:**
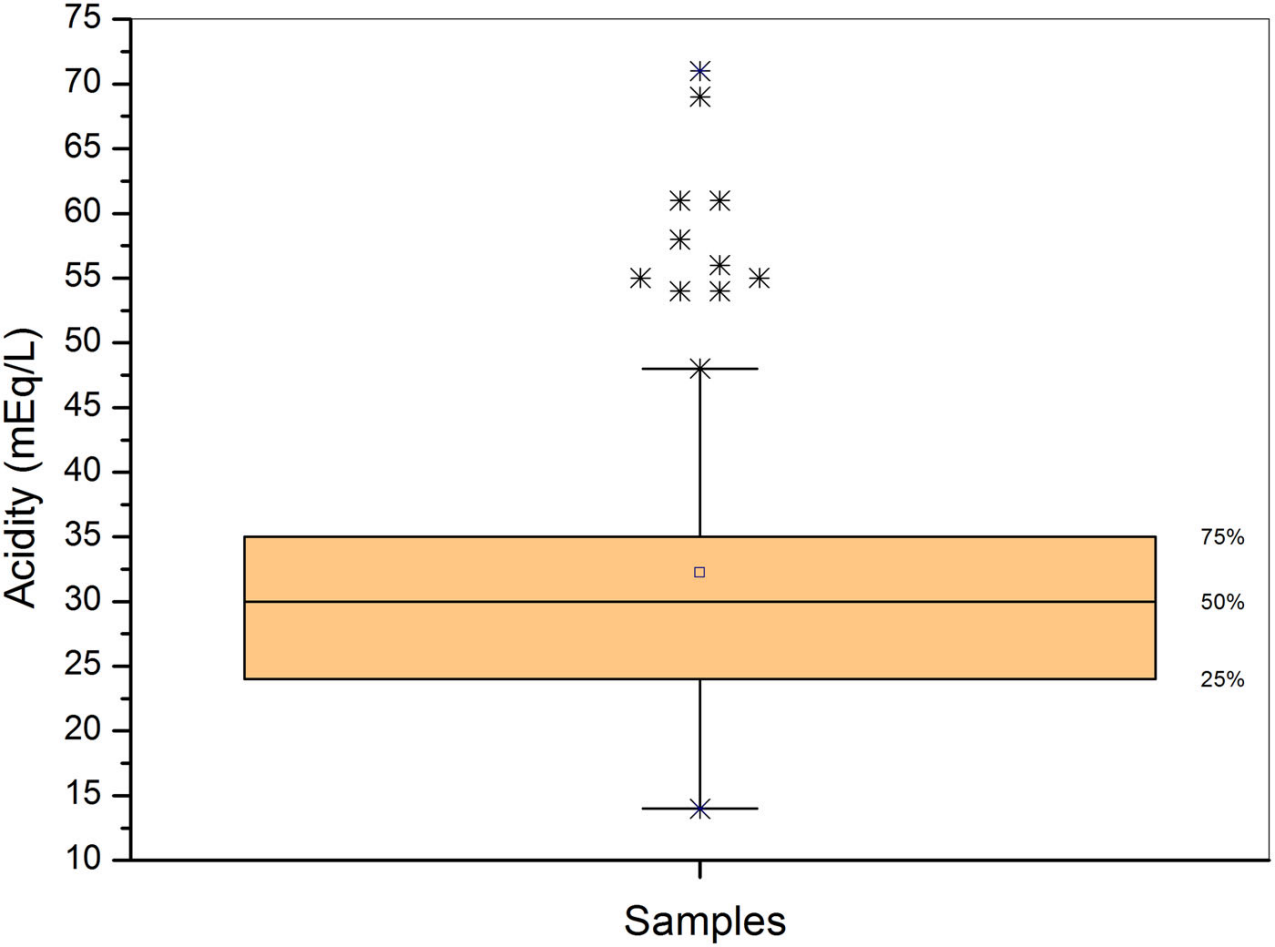
Boxplot of the acidity (percentage volume per volume [% v/v]) of commercial kombucha samples with values above the method's limit of quantification (LOQ). The square represents the median, asterisks represent outliers values.

The acidity levels found ranged from 17 to 71 mEq/L, as shown in the boxplot graph in Figure 3. Half of the samples had acidity between 24 and 35 mEq/L. Of the 96 samples analyzed, only 48 were within the range stipulated by legislation (30–130 mEq/L), with 38 samples below this range and 10 below the LOQ of the GC‐FID method. The median in the boxplot suggests a symmetric distribution, but nine outliers were identified with values above 48 mEq/L, highlighting the variability in the production of kombuchas.

Similar results were found in previous studies on national kombucha samples (Suhre 2020), highlighting ethanol levels ranging from 0.58% to 3.02% v/v, exceeding Brazil's regulatory limit for non‐alcoholic beverages. Additionally, a notable variation in the acidity of the samples was observed, encompassing values from 1.2 to 5.8 g/L (20–97 mEq/L).

A study conducted in the United States (Talebi et al. 2017) identified ethanol levels between 1.12% and 2.00% v/v in 18 commercial kombuchas, observing an increase correlated with storage time. The ethanol concentration in two batches of kombucha was monitored over 60 days under two different conditions: 4°C and 22°C. Furthermore, it was observed that with extended storage time, ethanol concentrations increased both in samples kept refrigerated and those stored at room temperature. This increase may be attributed to the presence of sugar and active yeasts, as well as the hydrolysis of ethyl esters.

The significant variation in the alcohol and acidity levels of the samples suggests discrepancies in production methods, such as the concentration of tea, sugar, flavoring ingredients, fermentation, and the composition of the SCOBY. These variables, along with storage conditions, influence fermentation and the properties of the final product (Watawana et al. 2015). The lack of compliance in labeling can pose health risks to consumers and expose producers to legal liability, in accordance with Resolution No. 432, dated January 23, 2013, from the National Traffic Council (Brasil 2013), also known as the “Zero Tolerance Law for Blood Alcohol,” which regulates the presence of alcohol in the blood of drivers in Brazil.

### 3.4 Development of PLS Models

The infrared transmission spectra of the 96 kombucha samples were obtained and are presented in Figure S3. Due to the high water content in the composition of the samples, it is difficult to identify the characteristic bands of the samples because of overlap with the broad bands typical of the water molecule. However, the main differences are observed in the regions from 3000 to 2250 cm^−1^ and 1500 to 750 cm^−1^.

#### 3.4.1 PLS Model for Determining Alcohol Content in Kombuchas Using Mid‐Infrared Spectroscopy

A multivariate calibration model using PLS regression was developed for the determination of alcohol content in kombucha samples, using as reference values the data obtained by GC‐FID. PLS regression is a multivariate technique widely used to model relationships between a set of independent variables (such as spectral data) and a dependent variable (such as alcohol content). To achieve this, the data are transformed into a new set of variables called latent variables (LVs), which concentrate most of the relevant information from both *X* (spectral data) and *y* (reference values) (ASTM International 2012).

A key step in developing the PLS model is selecting the optimal number of LVs. This selection is carried out through cross‐validation, a resampling technique that evaluates model performance using subsets of the data. The criterion used for this selection is the minimization of the root mean square error of cross‐validation (RMSECV), calculated by Equation (1):

(1) $$RMSECV=\sqrt{\frac{\sum_{i=1}^{n}{\left(y_{p}-y_{r}\right)}^{2}}{n}}$$

where *n* is the total number of samples in the cross‐validation set, *yp* is the predicted *y* value, and *yr* is the reference *y* value.

A total of 96 samples were used in the study, and the spectral range used was from 4000 to 900 cm^−1^, excluding the region from 901 to 550 cm^−1^ due to noise. The data were converted to absorbance using the equation *A* = log10 (1/*T*) (Barbosa 2011).

The samples were divided into calibration (64 samples) and validation (32 samples) sets using the Kennard–Stone algorithm, which ensures a representative distribution across the concentration range. To improve the quality of the spectral data, preprocessing was performed using the Savitzky–Golay first derivative (order 2; window of 15 points), followed by mean centering. The application of the Savitzky–Golay first derivative helps to smooth the noise and enhance important spectral features, while mean centering adjusts the data by removing the bias, making it easier to compare different samples. Cross‐validation was carried out using the random subset technique, with seven splits and 20 iterations, and the optimal number of LVs was determined based on the lowest RMSECV value obtained.

During the outlier detection step, 10 samples were identified in the calibration set and three in the validation set, totaling 13 samples, which corresponds to approximately 13.5% of the total and remains within the acceptable limit of 22%. After removing the outliers and re‐optimizing the model, the final model, with three LVs, was able to explain 92.23% of the variance in the spectral matrix *X* and 94.11% of the variance in the reference values in *Y*.

The model's performance parameters are presented in Table 2, which shows a significant improvement in the results after the exclusion of outliers. 

**TABLE 2:** PLS model parameters for determining the alcoholic content before and after outlier removal.

Multivariate calibration parameters		
	Before	After
Number of calibration samples	64	54
Number of validtion samples	32	29
Number of latent variables	3	3
RMSEC RMSECV	0.23 0.28	0.09 0.10
RMSEP *R* ^2^_cal_	0.24 0602	0.07 0938
*R* ^2^_val_	0521	0943

Abbreviations: *R* ^2^ cal = coefficient of determination for calibration; *R* ^2^ val = coefficient of determination for validation; RMSEC = root mean square error of calibration; RMSECV = root mean square error of cross‐validation; RMSEP = root mean square error of prediction.

**TABLE 3:** PLS model parameters for determining the acidity before and after outlier removal.

Multivariate calibration parameters		
	Before	Depois
Number of calibration samples	117	115
Number of validtion samples	59	51
Number of latent variables	2	2
RMSEC RMSECV	3.3 11.0	3.2 11.2
RMSEP	7.3	3.9
*R* ^2^_cal_ *R* ^2^_val_	0988 0932	0989 0982

Abbreviations: *R* ^2^ cal = coefficient of determination for calibration; *R* ^2^ val = coefficient of determination for validation; RMSEC = root mean square error of calibration; RMSECV = root mean square error of cross‐validation; RMSEP = root mean square error of prediction.

The quality of the developed model was assessed using the root mean square errors of calibration (RMSEC) and prediction (RMSEP), calculated for the calibration and external validation sets, respectively. RMSEC indicates the degree of scatter around the regression line in the calibration set, reflecting how well the model fits the experimental data. RMSEP, on the other hand, measures the model's accuracy in predicting new samples and serves as a direct indicator of its predictive performance.

**FIGURE 4:**
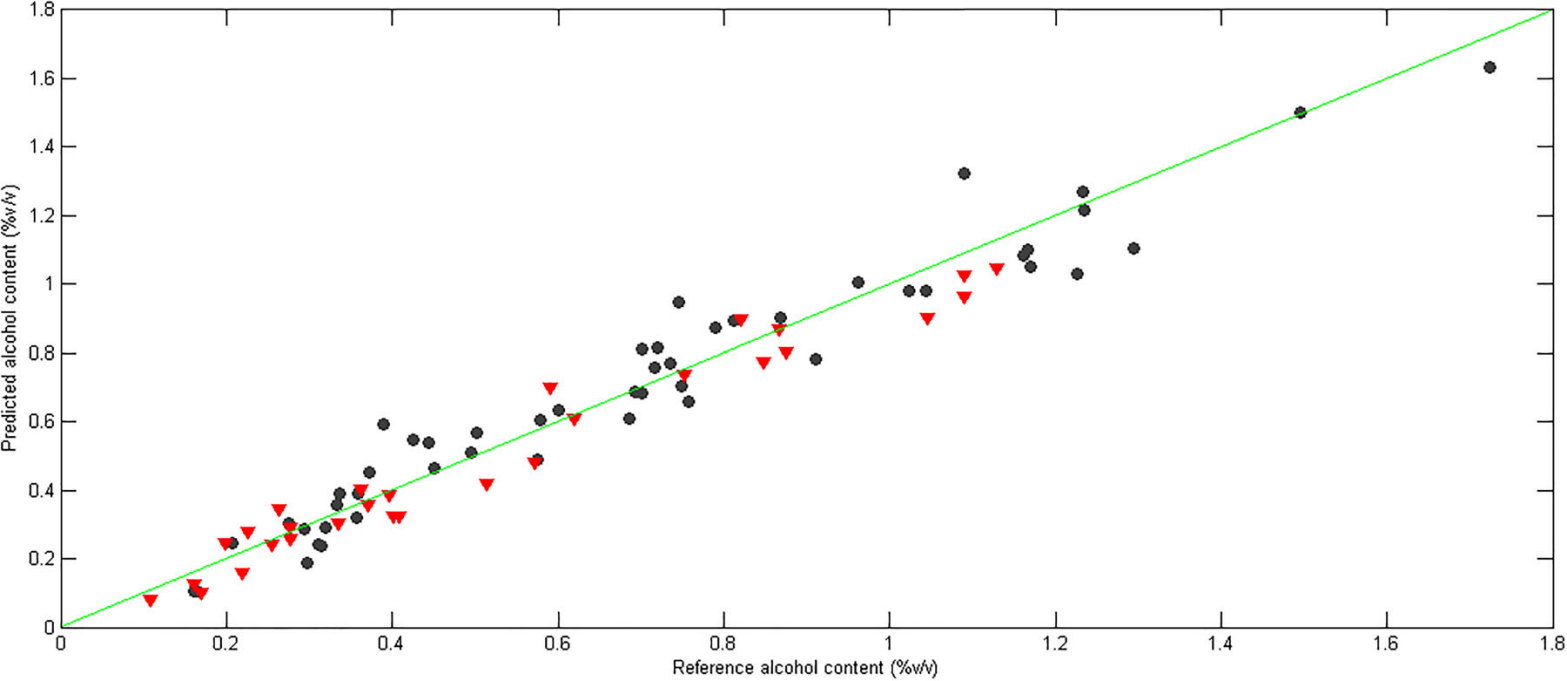
Plot of actual values (black circles) versus predicted values (red triangles) of alcoholic content (percentage volume per volume [% v/v]).

Both errors can be calculated using Equation (1), with the only difference being the dataset to which they are applied (calibration or validation). The RMSEC and RMSEP values obtained showed good accuracy, demonstrating the model's efficiency. Furthermore, the correlation coefficients were greater than *R* ^2^ > 0.9. This parameter assesses the correlation between the reference values and the predicted values in the calibration and validation sets, with values closer to 1 indicating a stronger correlation between the data (Fulgêncio et al. 2022; Botelho et al. 2013). This correspondence is illustrated in Figure 4, which presents a graphical comparison between the experimental values and those estimated by the model for the alcohol content.

**FIGURE 5:**
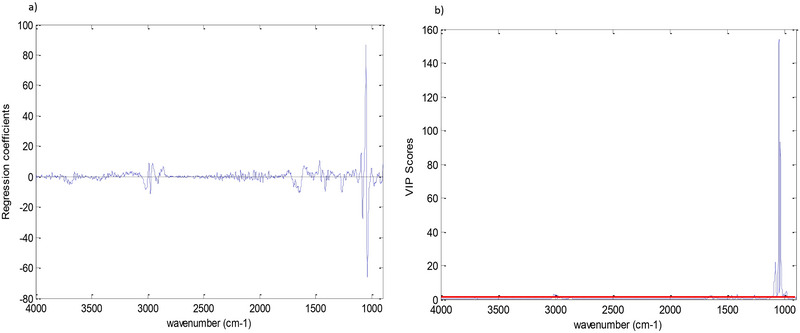
(a)Regression vector plot for *Y* versus wavenumber for the kombucha alcoholic content determination model. (b) Variable importance in projection (VIP) scores plot of *Y* versus wavenumber for the kombucha alcoholic content determination model.

The regression coefficients presented in Figure 5a illustrate the relative importance and direction (positive or negative correlation) of each spectral region in the prediction of alcohol content. The VIP (variable importance in projection) scores shown in Figure 5b indicate the overall relevance of each variable in building the model, with higher scores representing more significant variables for prediction. Spectral regions with VIP scores >1 are considered the most influential in the model. By analyzing Figure 5a,b together, it can be observed that the spectral regions with the greatest influence on the prediction of alcohol content are around 1050 cm⁻¹, corresponding to the C–O stretching vibration in primary alcohols (Barbosa 2011).

The model was validated in terms of merit figures: accuracy, precision, linearity, working range, bias, and the RPD, and the results are available in Table S2. The independence of residuals was evaluated using the Durbin–Watson test. RPD values above 2.4 suggest that the method has good predictive capacity (Fulgêncio et al. 2022; Botelho et al. 2013). The method's precision was also assessed through RSD for sextuplicates of three samples at three different levels, which were chosen to represent low, medium, and high alcohol contents, respectively.

After validation, the model demonstrated good predictive capacity with an RPD_val_ of 5.1 and an RPD_val_ of 3.4, as well as RSD values for repeatability and intermediate precision < 5%. The bias test revealed that the systematic errors present in the multivariate model are not statistically significant, which demonstrates the reliability of the results (assessed by a Student's *t*‐test with a 95% confidence level and 28 degrees of freedom) (Valderrama et al. 2009). The analysis of the alcohol content in the kombucha samples revealed an average value of 0.64% v/v, above the regulatory limit of 0.5% v/v for non‐alcoholic beverages (Brasil 2019), with this value being declared with an accepted tolerance of 0.1% v/v. The developed model presented an RMSEP of 0.07% v/v, allowing for the rapid quantification of this parameter in accordance with the legislation.

#### 3.4.2 PLS Model for Determining the Acidity of Kombuchas

A model was developed to determine the volatile acidity of kombuchas using reference values obtained by the GC‐FID method. 86 commercial samples with acidity above the LOQ of the GC‐FID method, ranging from 17 to 72 mEq/L, were used. However, since many of the samples had acidity concentrations between 0.1% and 0.2% v/v (corresponding to 17–35 mEq/L), to expand the measurement range, 10 samples were selected and spiked with acetic acid. These samples were spiked at nine different concentrations (0.10%, 0.15%, 0.20%, 0.25%, 0.30%, 0.35%, 0.40%, 0.50%, and 0.60% v/v), totaling 90 spiked samples. By combining the 90 spiked samples with the 86 commercial samples, a total of 176 samples were used to construct the model, with acidity concentrations ranging from 17 to 130 mEq/L. This approach allowed covering the entire range required by Brazilian legislation, which establishes limits between 30 and 130 mEq/L (Brasil 2019).

A spectral range used was from 4000 to 900 cm^−1^, excluding the region from 901 to 550 cm^−1^ due to noise. The data were converted to absorbance using the equation *A* = log10 (1/*T*) (Barbosa 2011).

**FIGURE 6:**
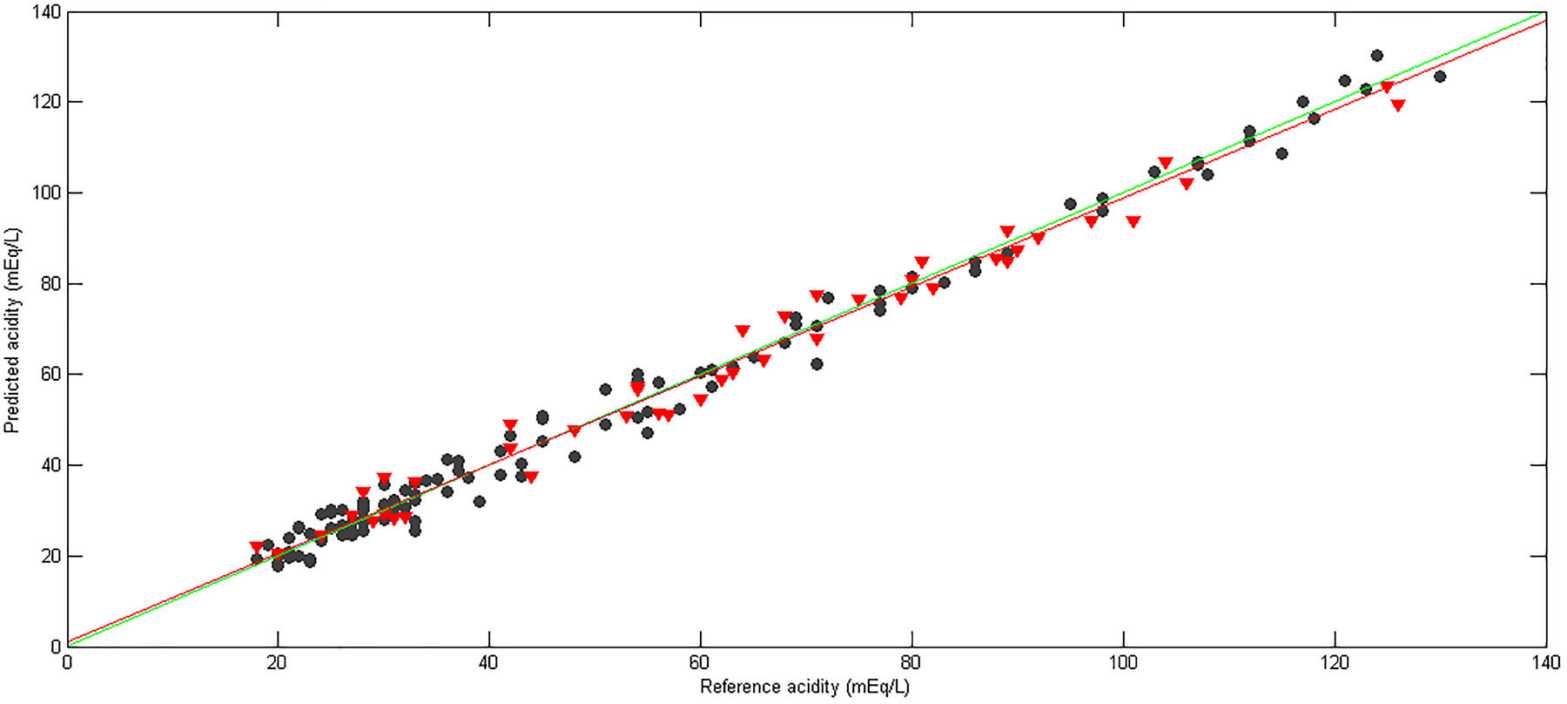
Plot of actual values (black circles) versus predicted values (red triangles) of acidity expressed as milliequivalents per liter (mEq/L).

A total of 117 samples were selected for the calibration set and 59 for the validation set using the Kennard–Stone algorithm. The data were preprocessed using orthogonal signal correction (OSC) and centered on the mean. Cross‐validation was performed using random subsets with 10 divisions and 20 iterations. The number of LVs was determined based on the lowest RMSECV. After detecting outliers, the model with two LVs explained 74.54% of the variance in *X* and 98.83% in *Y*. The model parameters indicate good accuracy and fit, as illustrated in Figure 6, which shows the real versus predicted acidity values.The model's performance parameters for acidity are presented in Table 3, which shows a significant improvement in the results after the exclusion of outliers.​

**FIGURE 7:**
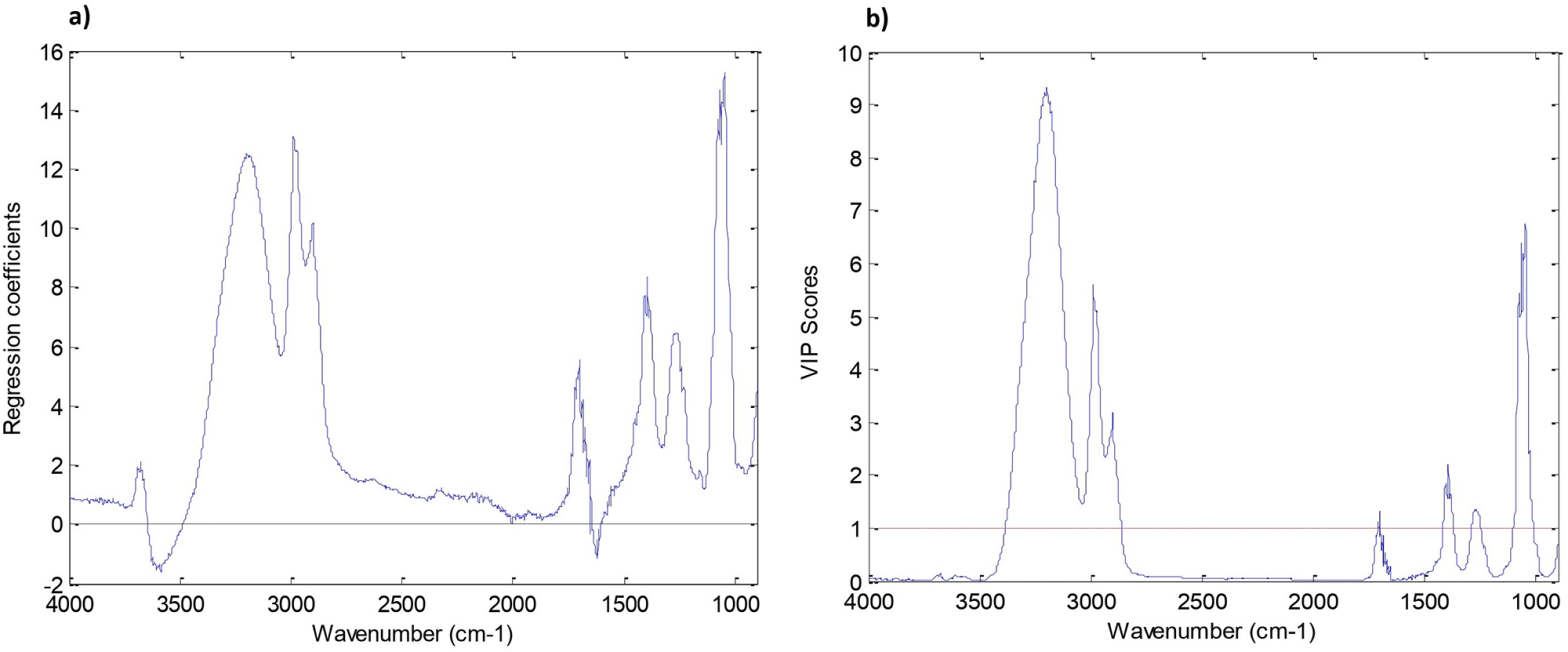
(a) Regression vector plot for *Y* versus wavenumber for the kombucha acidity determination model. (b) Variable Importance in Projection (VIP) Scores plot of *Y* versus wavenumber for the kombucha acidity determination model.

The graphs in Figure 7 indicate that the most relevant bands for the model are between 1300 and 1100 cm^−1^ (stretching of the C–O bond of acids), 1320–1210 cm^−1^ (angular deformation of C–O, appearing as a doublet for carboxylic acid dimers), and 1725–1700 cm^−1^ (stretching of the C = O bond of carboxylic acid in dimer form). A broad band of O–H bond stretching for carboxylic acid dimers is also highlighted, situated between 3300 and 2500 cm^−1^ (Barbosa 2011). Carboxylic acid molecules tend to bind through hydrogen bonds, forming a dimer. In this configuration, two molecules behave as a single molecule, with double the molecular mass (Barbosa 2011).

The chemometric model was validated, as shown in Table S3, presenting calibration and validation RPD values greater than 2.4 and RSD values for repeatability and intermediate precision below 5%. The bias test indicated that the systematic errors are not statistically significant, confirming the reliability of the model. Based on the volatile acidity results of the kombucha samples obtained, it was observed that the developed model allows for the quantification of this parameter with an approximate error of 11%, considering that the average acidity of the samples was 35 mEq/L and the RMSEP was 3.9 mEq/L.

## 4 Conclusions

The chemometric models developed in this study proved effective for monitoring the quality of commercial kombuchas, enabling the simultaneous quantification of alcohol content and volatile acidity in a fast manner with minimal sample preparation. The RMSEC, RMSEP, and *R* ^2^ values indicated a good model fit. After method validation, RPD values above 2.4 and precision with deviations below 5% were obtained, confirming the robustness and predictive capability of the models, even in complex matrices such as kombucha.

This study provides a comprehensive and representative overview of the kombucha market in Brazil, based on the analysis of 96 samples from 30 manufacturers located in different states and offering various flavor profiles. This approach enabled the identification of significant inconsistencies in product labeling and standardization, with direct implications for consumer protection and compliance with current legislation. The alcohol content of the samples ranged from 0.10% to 1.70% v/v, with 60% exceeding the regulatory limit of 0.5% v/v for non‐alcoholic beverages, highlighting the need for proper labeling. In terms of acidity, values ranged from 17 to 71 mEq/L, with some samples falling below the recommended specifications (30–130 mEq/L), and only 50% meeting the established parameters.

From a practical standpoint, the chemometric models developed represent an efficient and cost‐effective alternative to traditional methods such as chromatography. These models can be applied in food industry quality control laboratories, regulatory agencies, and research institutions, and they are aligned with the principles of green chemistry. The reduction in the use of chemical reagents, shorter analysis time, and operational simplicity make this approach especially advantageous for large‐scale routine analyses.

Furthermore, the data generated by this study provide valuable contributions to Brazilian regulatory agencies, such as ANVISA (National Health Surveillance Agency) and MAPA (Ministry of Agriculture, Livestock, and Supply), in the process of reviewing policies and inspection criteria. The findings also support producers in adjusting their production processes, contributing to enhanced food safety and greater transparency in the availability of these products to consumers.

## Author Contributions

**Gabriela Fioravante da Silva**: Conceptualization, data curation, formal analysis, investigation, validation, visualization, writing–original draft. **Bruno Gonçalves Botelho**: conceptualization, data curation, investigation, methodology, resources, supervision, project administration, funding acquisition, writing–review and editing.

## Conflicts of Interest

The authors declare no conflicts of Interest.

## Supporting information



Supplementary data to this article can be found online at
