# Supplementary material for: Simultaneous Quantification of Alcoholic Content and Acidity in Brazilian Commercial Kombuchas by FTIR Spectroscopy and Chemometric Analysis
Source: J Food Sci. 2025 Aug 3;90(8):e70433. doi: 10.1111/1750-3841.70433 (PMC12319122; doi:10.1111/1750-3841.70433)
Supplement: Supplementary file 1 — Supplementary data to this article can be found online at [file JFDS-90-0-s001.docx]

Supplementary data

Table S1: Statistical Analysis

| **Quality parameters** | **N total** | **Mean** | **Standard Deviation** | **Minimum** | **Median** | **Maximum** | **CI mean (95%)** |
| --- | --- | --- | --- | --- | --- | --- | --- |
| Alcoholic content (%v/v) | 96 | 0.64 | 0.36 | 0.10 | 0.60 | 1.70 | [0,57; 0,71] df=95 |
| Acidity (mEq/L) | 86 | 35 | 12 | 17 | 30 | 71 | [30; 36] df=85 |

Note: Of the total 96 available samples, only 86 showed acidity concentrations above the quantification limit established by the analytical method. CI: confidence interval of the mean; df: degrees of freedom.

 Figure S1: Evaluation of the linearity of the alcohol content using the gas chromatography with flame ionization detection (GC-FID) method. Black squares represent the solvent calibration curve, and red circles represent the matrix-matched calibration curve. A/IS: analyte to internal standard ratio.

 Figure S2: Evaluation of the linearity of acidity using the gas chromatography with flame ionization detection (GC-FID) method. Black squares represent the solvent calibration curve, and red circles represent the matrix-matched calibration curve. A/IS: analyte to internal standard ratio.

 Figure S3: Mid-infrared (MIR) spectra of 96 kombucha samples. Source: Fourier transform infrared (FTIR) spectrophotometer, Frontier model, PerkinElmer brand.

Table S2: Validation merit figures for the PLS model for determining the alcoholic content of kombucha.

| **Figure of merit** | **Parameter** | **Value** |
| --- | --- | --- |
| Precision | RSD repeatability (%) | 3,6 - 4,2 |
|  |  |  |
|  | RSD intermediate precision (%) | 3,8 – 4,7 |
| Linearity | Durbin–Watson test  parameter | d=1,80  dl (α=0,05)=1,34  du (α=0,05)=1,48 |
|  | Slope | 0,95±0,02 |
|  | Intercept | 0,024±0,018 |
|  | Correlation coefficient | 0,9478 |
|  | Working range | 0,16 -1,72 %v/v |
| Bias | Bias | - 0,03 |
|  | t _calculated_ | 1,803 |
|  | t _critical (28;95%)_ | 2,048 |
| RPD | Calibration set | 3,4 |
|  | Validation set | 5,1 |

Source: Prepared by the author. Legend: RSD = Relative Standard Deviation; RPD = Ratio of Performance to Deviation.Notes: Durbin-Watson test: if d > dl and du, the residuals are independent. T-test: if tcal < tcri, the systematic errors are not statistically significant.

Table S3: Validation merit figures for the PLS model for determining the acity of kombucha.

| **Figure of merit** | **Parameter** | **Value** |
| --- | --- | --- |
| Precision | RSD repeatability (%) | 3,2 – 4,3% |
|  |  |  |
|  | RSD intermediate precision (%) | 3,8 – 4,6% |
| Linearity | Durbin–Watson test  parameter | d=1,98  dl (α=0,05)=1,46  du (α=0,05)=1,55 |
|  | Slope | 0,98±0,01 |
|  | Intercept | 1,04±0,56 |
|  | Correlation coefficient | 0,9868 |
|  | Working range | 18 -130 mEq/L |
| Bias | Bias | 0,286 |
|  | t_cal_ | 0,462 |
|  | T_crit (50;95%)_ | 2,009 |
| RPD | Calibration set | 3,7 |
|  | Validation set | 5,2 |

Source: Prepared by the author. Legend: RSD = Relative Standard Deviation; RPD = Ratio of Performance to Deviation.Notes: Durbin-Watson test: if d > dl and du, the residuals are independent. T-test: if tcal < tcri, the systematic errors are not statistically significant.
